# Data on the role of accessible surface area on osmolytes-induced protein stabilization

**DOI:** 10.1016/j.dib.2016.11.055

**Published:** 2016-11-23

**Authors:** Safikur Rahman, Syed Ausaf Ali, Asimul Islam, Md. Imtaiyaz Hassan, Faizan Ahmad

**Affiliations:** Centre for Interdisciplinary Research in Basic Sciences, Jamia Millia Islamia, New Delhi 110025, India

**Keywords:** Osmolytes, Methylamines, Protein stabilization, Accessible surface area, Gibbs free energy

## Abstract

This paper describes data related to the research article “Testing the dependence of stabilizing effect of osmolytes on the fractional increase in the accessible surface area on thermal and chemical denaturations of proteins” [1]. Heat- and guanidinium chloride (GdmCl)-induced denaturation of three disulfide free proteins (bovine cytochrome *c* (b-cyt-*c*), myoglobin (Mb) and barstar) in the presence of different concentrations of methylamines (sarcosine, glycine-betaine (GB) and trimethylamine-N-oxide (TMAO)) was monitored by [*ϴ*]_222_, the mean residue ellipticity at 222 nm at pH 7.0. Methylamines belong to a class of osmolytes known to protect proteins from deleterious effect of urea. This paper includes comprehensive thermodynamic data obtained from the heat- and GdmCl-induced denaturations of barstar, b-cyt-*c* and Mb.

**Specifications Table**

**Table:** 

Subject area	Chemistry
More specific subject area	Protein chemistry
Type of data	Tables, figures
How data were acquired	Experiments were performed using Jasco spectropolarimeter, Model J-1500-150 (JASCO Corporation, Japan), equipped with Peltier-type temperature controller
Data format	Raw, Plotted, analyzed
Experimental factors	All samples and buffers were filtered with 0.22 μm Millipore filters and degassed.
Experimental features	All CD spectra were recorded at 1 nm band width, temperature scan rate 1 °C/min and data was collected at every 0.1 °C
Data source location	Jamia Millia Islamia, New Delhi, India
Data accessibility	Data are accessible in this article

**Value of the data**

• Methylamines are stabilizing osmolytes. That is, they shift midpoint of denaturation curves to higher *C*_m_ (midpoint of the GdmCl-induced unfolding transition) and *T*_m_ (midpoint of the heat-induced unfolding transition). *C*_m_ and *T*_m_ increase with increase in concentrations of methylamines.
• Stabilization effect of methylamines in terms of $\Delta G_{D}^{o}$ (Gibbs free energy change) obtained from GdmCl-induced denaturation studies are found to be more than that from thermal transitions in cases of Mb and barstar.

• The stabilizing effect of methylamine against heat- and GdmCl-induced denaturation is same in the case of b-cyt-*c*.

## 1 Data

Heat- and GdmCl-induced transition curves of proteins were monitored by [*ϴ*]_222_ measurements. These transition curves were analyzed for thermodynamic parameters according to Eqs.(1), (2), (3), (4).

**Fig. 1:**
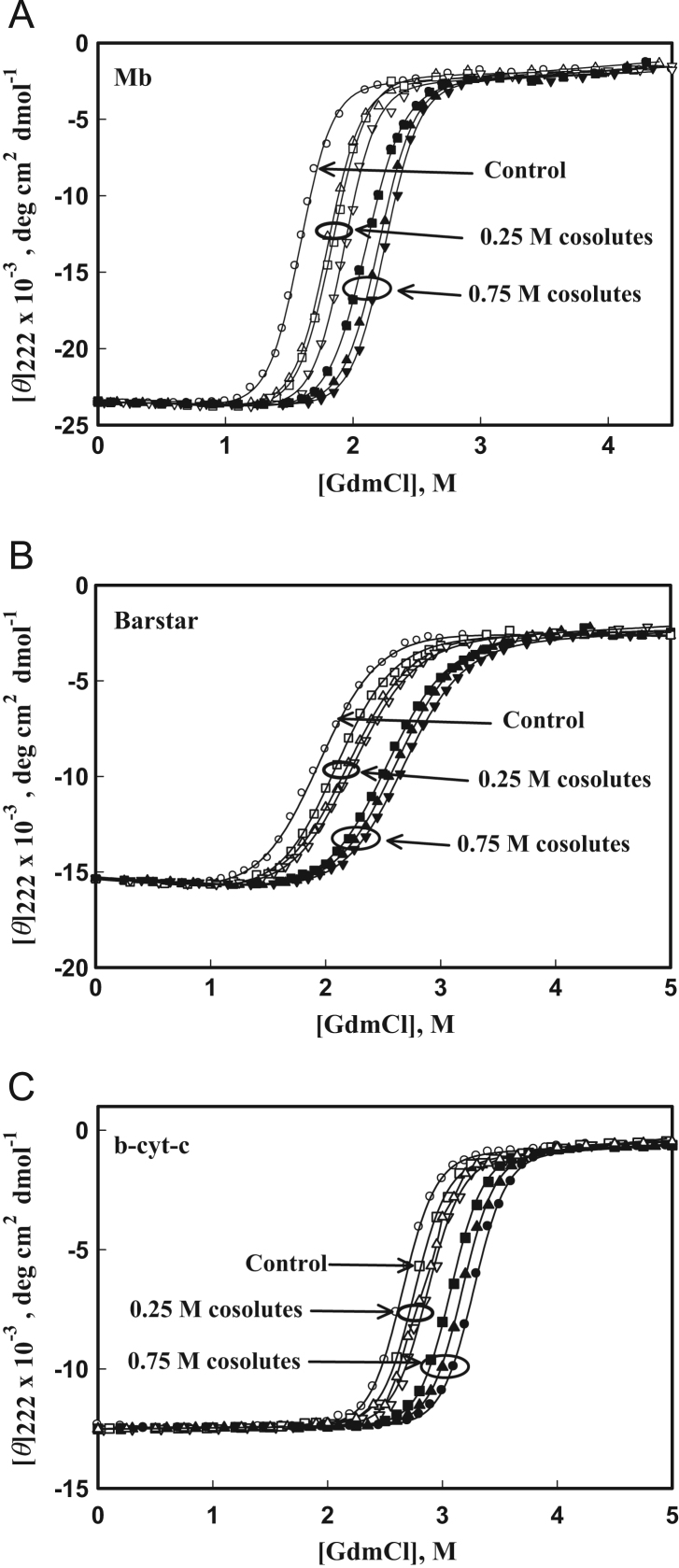
GdmCl-induced denaturation curves of proteins: GdmCl-induced denaturation curves of Mb, barstar and b-cyt-*c* in the presence of 0.25 and 0.75 M osmolytes at pH 7.0 and 25 ^o^C: control (○) represents denaturation curve in the absence of osmolytes. Symbols (Δ), (∇) and (▢) represent 0.25 M sarcosine, 0.25 M TMAO and 0.25 MGB, respectively, while (▲), (▼) and (■) represent 0.75 M TMAO, 0.75 M sarcosine and 0.75 MGB, respectively. To maintain clarity all data points are not shown.

We have carried out GdmCl- and heat-induced denaturation experiments of barstar, b-cyt-*c* and Mb in the absence and presence of different concentrations of different methylamine by following the change in [*ϴ*]_222_ (probe for measuring change in secondary structure). Fig. 1 shows GdmCl-induced denaturation curves of Mb, barstar and b-cyt-*c* in the absence and presence of 0.25 and 0.75 M of each of sarcosine, glycine-betaine and TMAO at pH 7.0 and 25 °C. Denaturation of each of protein was found to be reversible in entire range of methylamine concentrations. Each transition curve was measured at least three times, and analyzed for thermodynamic parameters using the Eq. (1). Values of $\Delta G_{D}^{o}$, *m*_g_ and *C*_m_ thus obtained are given elsewhere [1].

**Fig. 2:**
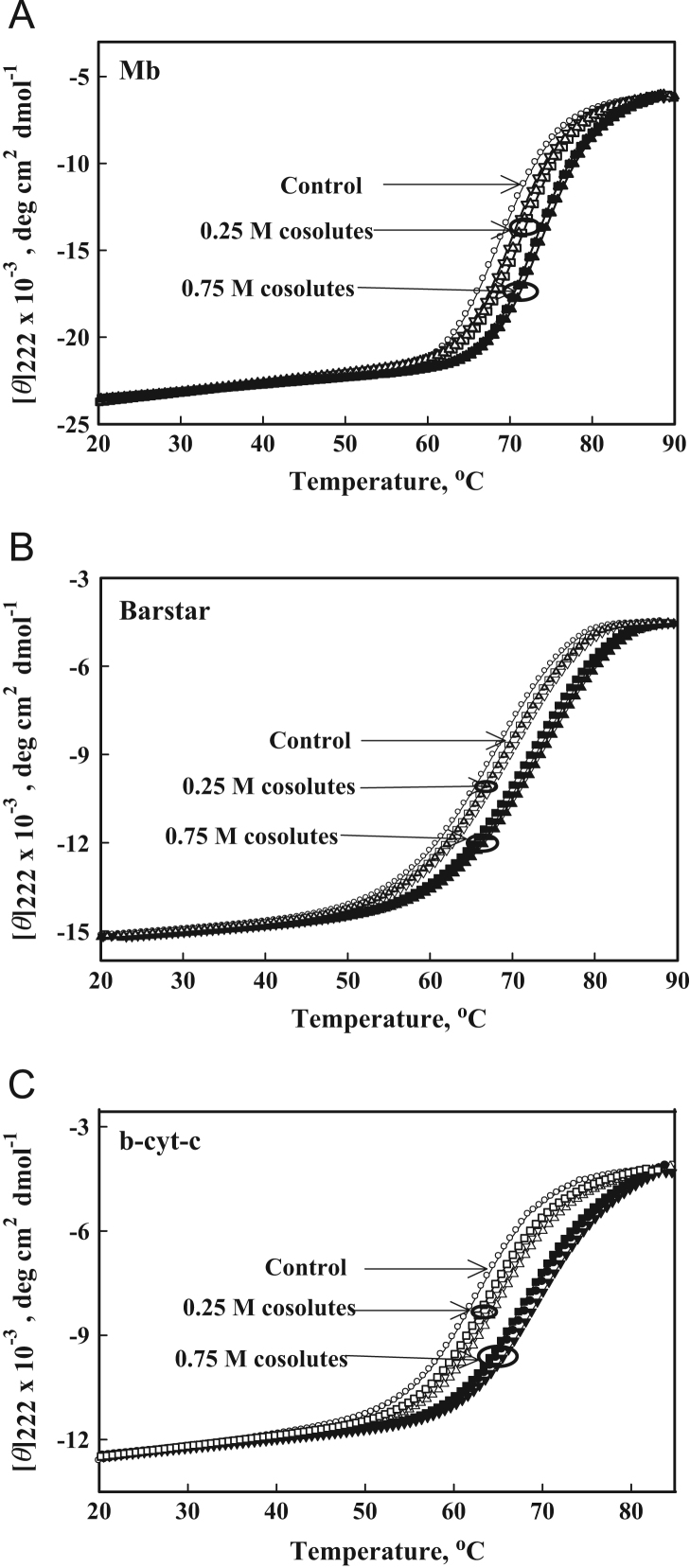
Heat-induced denaturation curves of proteins at pH 7.0: Heat-induced denaturation curves of Mb, barstar and b-cyt-*c* in the presence 0.25 and 0.75 M osmolytes at pH 7.0: Denaturation curves in cases of Mb and b-cyt-*c* were obtained in the presence of 0.6 and 1.25 M GdmCl, respectively. Symbols have same meaning as in Fig. 1.

**Fig. 3:**
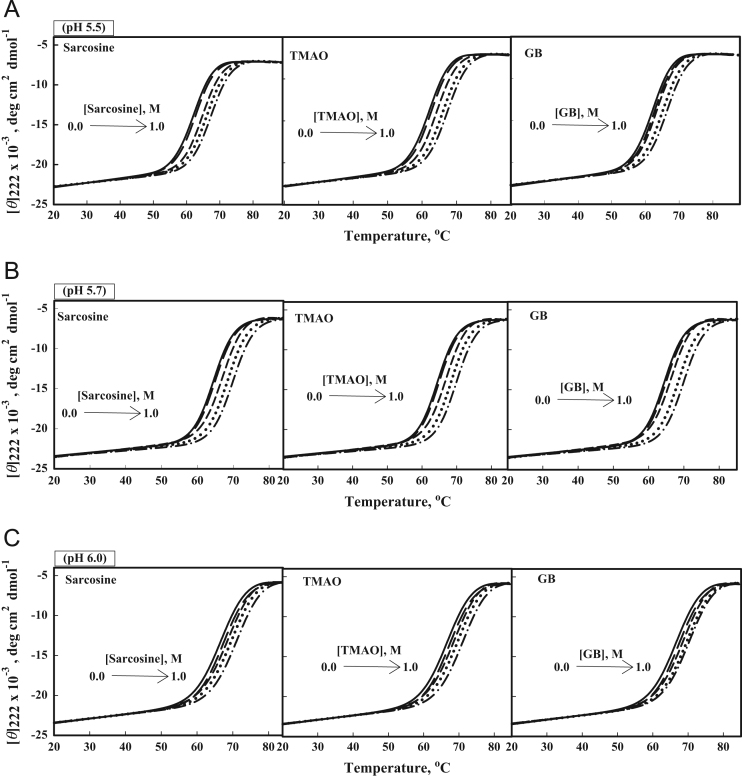
Heat-induced denaturation of Mb: Heat-induced denaturation curves of Mb in the absence and presence of 0, 0.25, 0.5, 0.75 M and 1.0 M osmolytes: (A) Sarcosine, TMAO and GB at pH values 5.5; (B) Sarcosine, TMAO and GB at pH values 5.7; and (C) Sarcosine, TMAO and GB at pH values 6.0. Lines (solid line), (long dash), (short dash), (dotted) and (dash-dot) represent 0.00, 0.25, 0.50, 0.75 and 1.00 M of each of co-solute, respectively. These denaturation curves were obtained in the presence of 0.6 GdmCl.

**Fig. 4:**
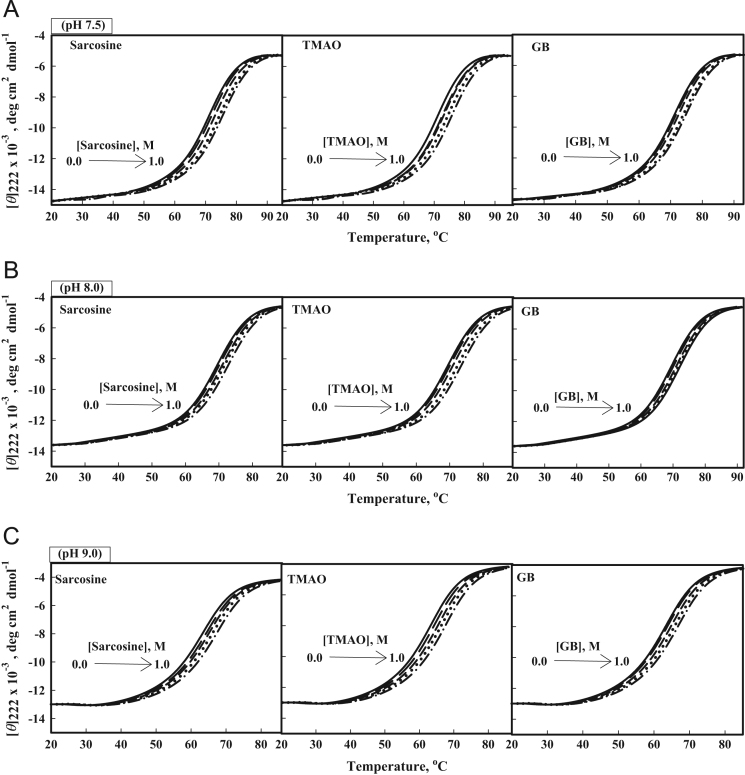
Heat-induced denaturation of barstar: Heat-induced denaturation curves of barstar in the absence and presence of 0, 0.25, 0.5, 0.75 M and 1.0 M osmolytes: (A) Sarcosine, TMAO and GB at pH values 7.5; (B) Sarcosine, TMAO and GB at pH values 8.0; and (C) Sarcosine, TMAO and GB at pH values 9.0. Lines have same meaning as in Fig. 3.

**Fig. 5:**
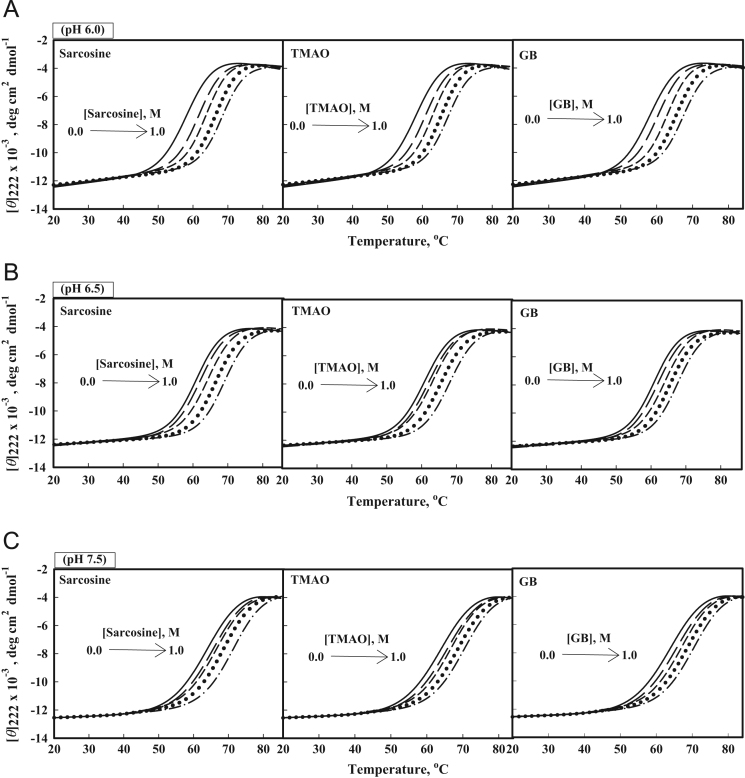
Heat-induced denaturation of b-cyt-*c*: Heat-induced denaturation curves of b-cyt-*c* in the absence and presence of 0, 0.25, 0.5, 0.75 M and 1.0 M osmolytes: (A) Sarcosine, TMAO and GB at pH values 6.0; (B) Sarcosine, TMAO and GB at pH values 6.5; and (C) Sarcosine, TMAO and GB at pH values 7.5. Lines have same meaning as in Fig. 3. These denaturation curves were obtained in the presence of 1.25 M GdmCl.

**Fig. 6:**
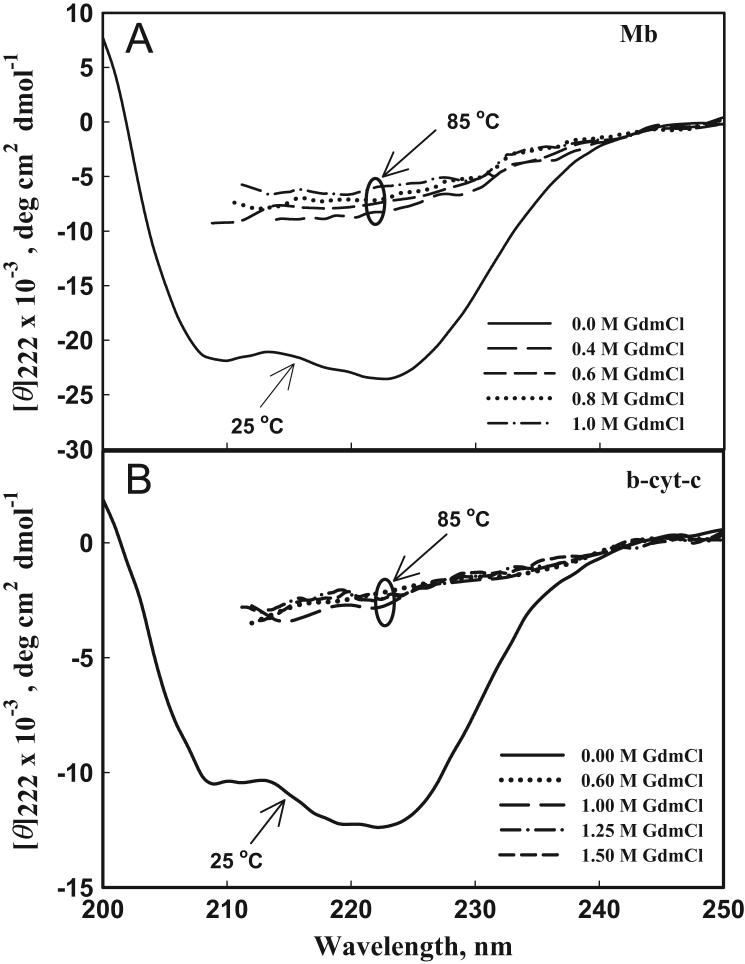
The far-UV CD spectra of Mb (A) and b-cyt-*c* (B) in the presence of different concentrations of GdmCl as indicated in the figure: For comparison of these spectra, the far-UV CD spectra of proteins in the absence of GdmCl at 25 ^o^C (native state) are also shown in this figure.

Fig. 2 shows heat-induced denaturation curves of Mb, barstar and b-cyt-*c* in the presence of 0, 0.25 and 0.75 M sarcosine, glycine-betaine and TMAO at pH 7.0. Furthermore, Fig. 3, Fig. 4, Fig. 5 show heat-induced denaturation curves of these proteins in the presence of 0.25, 0.5, 0.75 and 1.0 M of each methylamine (sarcosine, glycine-betaine and TMAO) at pH values other than 7.0. All these denaturation curves (Fig. 2, Fig. 3, Fig. 4, Fig. 5) were monitored by change in [*ϴ*]_222_ and were measured at least in triplicate. Thermal denaturation of each protein in the entire range of each [methylamine], the molar concentration of methylamine, was reversible at all pH values. It was observed that the temperature-dependence of *y*_N_, the optical property of the native (N) state of the protein depends on neither [methylamine] nor pH. However, *y*_D,_ the optical property of the denatured (D) state of the protein depends on pH (Fig. 2, Fig. 3, Fig. 4, Fig. 5). Each denaturation curve of the protein at given (methylamine) was analyzed for thermodynamic parameters, namely Δ*H*_m_, *T*_m_, Δ*C*_p_ and $\Delta G_{D}^{o}$ using Eqs. (2), (3), (4), and the values are given in Table 1, Table 2, Table 3 (values for pH 7.0 are given elsewhere [1]). Fig. 6 shows far-UV CD spectra of Mb and b-cyt-*c* in the absence and presence of different concentrations of GdmCl at 85 °C. It is seen in this figure that [*θ*]_222_ of Mb depends significantly on the (GdmCl). However, this dependence is insignificant in the case of b-cyt-*c*.

## 2 Experimental design, materials and methods

### 2.1 GdmCl-induced denaturation studies in the absence and presence of methylamines

GdmCl-induced transition between N and D states of b-cyt-*c*, Mb, and barstar in the absence and presence of different methylamines were monitored by [*ϴ*]_222_ at pH 7.0 and 25 °C. Using a non-linear least-squares method, the entire data (*y*(g), [g]) of each denaturant-induced transition curve were analyzed for ${\Delta G}_{D}^{o}$, *m*_g_ and *C*_m_ using the relation [2],

(1) $$y\left(g\right)=\frac{y_{N}\left(g\right)+y_{D}(g)\times e^{[-\left(∆G_{D}^{0}+m_{g}\left[g\right]\right)/\mathrm{RT}]}}{1+e^{[-\left(∆G_{D}^{0}+m_{g}\left[g\right]\right)/\mathrm{RT}]}}$$

where *y*(g) is the observed [*θ*]_222_ at [g], the molar concentration of GdmCl, *y*_N_ and *y*_D_ are [*θ*]_222_ values of N and D molecules under the same experimental conditions in which *y*(g) was measured, ${\Delta G}_{D}^{o}$ is the value of Gibbs free energy change in the absence of the denaturant, *m*_g_ is the slope (∂Δ*G*_D_/∂[g])_T,P_, *R* is the universal gas constant and *T* is the temperature in Kelvin. It should, however, be noted that the derivation of Eq. (1) assumes that GdmCl-induced denaturation of each protein is a two-state process. Another assumption is that [g]-dependencies of *y*_N_(g) and *y*_D_(g) are linear (i.e., *y*_N_(g)=*a*_N_+*b*_N_ [g] and *y*_D_(g)=*a*_D_+*b*_D_ [g], where *a* and *b* are [g]-independent parameters, and subscripts N and D represent these parameters for the native and denatured protein molecules, respectively.

### 2.2 Heat-induced denaturation studies in the presence and absence of osmolytes

Heat-induced denaturation of Mb, b-cyt-*c* and barstar in the absence and presence of different concentrations of each osmolyte (sarcosine, TMAO and glycine betaine) were monitored by [*θ*]_222_ at different pH values. Methods for determining the authentic values of thermodynamic parameters from the analysis of thermal denaturation curves of optical properties have already been published [3], [4], [5]. It should be noted that this analysis assumes that (i) the transition between N and D states of the protein in the absence and presence of each osmolyte is a two-state process, and (ii) structural characteristics of both N and D states are not affected by osmolytes. Each denaturation curve of the protein at a given [methylamine] and pH was analyzed for *T*_m_ and Δ*H*_m_ using a non-linear least-squares method that involves fitting the entire ([*θ*]_222_, *T*) data of the transition curve to Eq. (2) with all eight free parameters (a_N_, b_N_, c_N_, a_D_, b_D_, c_D_, *T*_m_ and Δ*H*_m_).

(2) $$y(T)=\frac{y_{N}(T)+y_{D}(T)\exp[-\Delta H_{m}/R(1/T-\;\text{1}/T_{m})]}{1+\exp[-\Delta H_{m}/R(1/T\;-\;\text{1}/T_{m})]}$$

where *y*(*T*) is the optical property at temperature *T* (Kelvin), *y*_N_(*T*) and *y*_D_(*T*) are the optical properties of the native and denatured protein molecules at temperature *T* (Kelvin) and *R* is the gas constant. As described earlier [3], [4], [5], in the analysis of the transition curve, it was assumed that a parabolic function describes the dependence of the optical properties of the native and denatured protein molecules (i.e., *y*_N_(*T*)=*a*_N_+*b*_N_*T*+*c*_N_*T*^2^, and *y*_D_(*T*)=*a*_D_+*b*_D_*T*+*c*_D_*T*^2^, where *a*_N_, *b*_N_, *c*_N_, *a*_D_, *b*_D_, and *c*_D_ are temperature-independent coefficients). The temperature-independent constant-pressure heat capacity change (Δ*C*_p_) was determined from slope of the linear plot of ∆*H*_m_ versus *T*_m_, using the relation:

(3) $${∆C}_{p}=\left(\frac{{\partial ∆H}_{m}}{{\partial T}_{m}}\right)p$$

Using values of *T*_m_, ∆*H*_m_ and Δ*C*_p_ the value of Δ*G*_D_ at any temperature *T*, ∆*G*_D_(*T*), was estimated with the help of Gibbs-Heltmholtz equation:

(4) $$\Delta G_{D}(T)=\Delta H_{m}\left(\frac{T_{m}\;-\;T}{T_{m}}\right)-\Delta C_{p}\left[(T_{m}-T)+T\;\ln\left(\frac{T}{T_{m}}\right)\right]\;$$

## Figures and Tables

**Table 1:** Thermodynamic parameters associated with the thermal denaturation of myoglobin in the absence and presence of sarcosine, TMAO and GB at different concentrations and pH values.

**[Osmolytes] M**	**pH 5.5**			**pH 5.7**			**pH 6.0**		
	${\Delta G}_{D}^{o}$** kcal mol**^**−1**^	***T***_**m**_(^**°**^**C)**	**∆*****H***_**m**_** kcal mol**^**−1**^	${\Delta G}_{D}^{o}$** kcal mol−**^**1**^	***T***_**m**_**(**^**°**^**C)**	**∆*****H***_**m**_** kcal mol**^**−1**^	${\Delta G}_{D}^{o}$** kcal mol**^**−1**^	***T***_**m**_(^**°**^**C)**	**∆*****H***_**m**_** kcal mol**^**−1**^
**Sarcosine**									
0.00	4.80±0.20	77.5±0.4	101±3	5.15±0.19	79.5±0.3	105±3	5.51±0.35	82.5±0.4	110±4
0.25	5.06±0.38	78.3±0.4	102±3	5.41±0.28	80.4±0.4	106±3	6.13±0.28	83.5±0.3	113±3
0.50	5.22±0.33	79.7±0.3	103±3	5.57±0.18	81.9±0.4	107±2	6.31±0.17	84.9±0.3	114±2
0.75	5.38±0.45	81.0±0.4	106±4	5.73±0.33	83.1±0.3	110±3	6.52±0.34	86.7±0.2	116±3
1.00	5.80±0.17	82.3±0.4	108±2	6.12±0.38	84.6±0.3	112±4	6.92±0.42	87.8±0.4	118±4
**TMAO**									
0.25	5.27±0.17	78.2±0.3	103±2	5.48±0.33	80.2±0.4	106±3	6.05±0.17	83.2±0.4	112±2
0.50	5.60±0.38	79.1±0.4	105±4	5.83±0.19	81.1±0.3	108±2	6.53±0.42	84.5±0.3	115±4
0.75	5.78±0.10	80.0±0.4	106±2	6.30±0.40	82.3±0.3	111±4	7.08±0.16	85.4±0.4	118±2
1.00	6.25±0.13	81.1±0.3	108±2	6.60±0.34	83.6±0.3	112±4	7.40±0.32	86.4±0.4	119±4
**GB**									
0.25	5.24±0.12	78.1±0.4	103±2	5.62±0.24	80.0±0.4	107±3	6.19±0.23	83.0±0.3	113±3
0.50	5.39±0.19	78.8±0.2	104±3	5.72±0.12	81.2±0.3	108±2	6.46±0.14	84.2±0.3	115±2
0.75	5.72±0.30	79.4±0.2	106±4	6.03±0.14	82.0±0.3	110±2	6.74±0.12	85.4±0.3	117±2
1.00	6.04±0.13	80.3±0.3	107±2	6.19±0.23	83.1±0.2	110±3	7.13±0.23	86.3±0.4	118±4

**Table 2:** Thermodynamic parameters associated with the thermal denaturation of b-cyt-*c* in the absence and presence of sarcosine, TMAO and GB at different concentrations and pH values.

**[Osmolytes] M**	**pH 6.0**			**pH 6.5**			**pH 7.5**		
	${\Delta G}_{D}^{o}$** kcal mol**^**−1**^	***T***_**m**_(^**°**^**C)**	**∆*****H***_**m**_** kcal mol**^**−1**^	${\Delta G}_{D}^{o}$** kcal mol**^**−1**^	***T***_**m**_(^**°**^**C)**	**∆*****H***_**m**_** kcal mol**^**−1**^	${\Delta G}_{D}^{o}$** kcal mol**^**−1**^	***T***_**m**_**(**^**°**^**C)**	**∆*****H***_**m**_** kcal mol**^**−1**^
**Sarcosine**									
0.00	9.51±0.39	80.4±0.5	97±3	9.95±0.23	84.3±0.4	99±2	11.28±0.22	89.1±0.3	106±3
0.25	9.82±0.17	82.5±0.4	98±2	10.42±0.29	85.2±0.4	101±4	11.60±0.10	90.6±0.3	107±3
0.50	10.49±0.18	84.4±0.4	102±3	10.93±0.40	87.4±0.3	104±3	12.11±0.34	91.1±0.2	110±2
0.75	10.95±0.29	86.1±0.3	103±4	11.71±0.34	89.7±0.3	107±3	12.74±0.15	93.2±0.2	112±3
1.00	11.64±0.23	87.9±0.5	106±3	12.69±0.22	92.3±0.4	111±2	13.50±0.26	94.8±0.3	115±4
**TMAO**									
0.25	9.85±0.23	83.0±0.5	98±3	10.24±0.29	85.1±0.4	100±4	11.59±0.10	90.5±0.3	107±3
0.50	10.30±0.35	85.0±0.4	100±4	10.67±0.34	86.4±0.4	102±4	11.87±0.34	92.0±0.4	108±4
0.75	10.92±0.24	87.4±0.4	103±4	11.34±0.41	88.7±0.5	105±5	12.31±0.10	92.5±0.4	111±3
1.00	11.57±0.22	89.1±0.3	106±3	12.18±0.20	91.3±0.4	109±3	13.00±0.38	93.9±0.5	113±5
**GB**									
0.25	9.78±0.12	82.2±0.4	98±2	10.53±0.45	85.1±0.4	102±4	11.70±0.22	90.1±0.3	108±3
0.50	10.25±0.21	83.9±0.4	100±3	11.02±0.12	86.6±0.4	104±3	12.05±0.16	91.3±0.2	109±2
0.75	10.97±0.34	85.4±0.4	102±4	11.60±0.17	88.4±0.3	105±3	12.64±0.15	92.2±0.2	110±3
1.00	11.31±0.17	86.9±0.5	104±3	12.07±0.18	90.0±0.4	108±2	12.99±0.33	93.6±0.3	112±4

**Table 3:** Thermodynamic parameters associated with the thermal denaturation of barstar in the absence and presence of sarcosine, TMAO and GB at different concentrations and pH values.

**[Osmolytes] M**	**pH 7.5**			**pH 8.0**			**pH 9.0**		
	${\Delta G}_{D}^{o}$** kcal mol**^**−1**^	***T***_**m**_**(**^**°**^**C)**	**∆*****H***_**m**_ **kcal mol**^**−1**^	${\Delta G}_{D}^{o}$** kcal mol**^**−1**^	***T***_**m**_**(**^**°**^**C)**	**∆*****H***_**m**_** kcal mol**^**−1**^	${\Delta G}_{D}^{o}$**kcal mol**^**−1**^	***T***_**m**_**(^°^C)**	**∆*****H***_**m **_**kcal mol**^**−1**^
**Sarcosine**									
0.00	4.05±0.16	69.4±0.2	61±3	3.58±0.20	66.0±0.3	57±4	3.02±0.24	62.0±0.3	52±3
0.25	4.51±0.22	70.2±0.2	64±2	3.92±0.16	66.9±0.3	59±3	3.42±0.17	63.0±0.2	55±2
0.50	4.78±0.18	71.3±0.4	66±3	4.19±0.18	67.6±0.2	61±3	3.51±0.21	63.5±0.2	56±3
0.75	5.19±0.16	72.2±0.3	69±2	4.52±0.24	68.7±0.4	64±4	3.87±0.19	64.4±0.3	59±2
1.00	5.55±0.21	73.1±0.2	72±2	4.86±0.21	69.8±0.3	67±4	4.19±0.18	65.1±0.4	62±3
**TMAO**									
0.25	4.39±0.22	70.3±0.3	63±2	3.82±0.19	66.8±0.3	59±3	3.33±0.19	63.1±0.2	54±3
0.50	4.69±0.23	70.8±0.2	66±3	4.06±0.16	67.6±0.2	60±2	3.58±0.27	63.8±0.3	56±2
0.75	4.93±0.21	71.5±0.2	67±3	4.31±0.26	68.5±0.3	63±2	3.70±0.21	64.2±0.3	58±3
1.00	5.30±0.24	72.7±0.3	70±2	4.57±0.24	69.4±0.4	65±4	3.94±0.18	65.0±0.2	60±4
**GB**									
0.25	4.30±0.17	70.0±0.3	63±3	3.75±0.22	66.7±0.3	58±4	3.24±0.21	62.8±0.2	54±2
0.50	4.56±0.21	70.9±0.2	65±2	3.94±0.18	67.4±0.2	60±3	3.47±0.19	63.5±0.3	56±2
0.75	4.79±0.25	71.0±0.3	65±3	4.20±0.25	68.3±0.3	62±3	3.75±0.18	64.0±0.2	57±4
1.00	5.11±0.24	72.1±0.2	70±2	4.40±0.27	69.2±0.2	64±2	3.94±0.23	65.2±0.2	61±3
